# Relationship between Volatile Composition and Bioactive Potential of Vegetables and Fruits of Regular Consumption—An Integrative Approach

**DOI:** 10.3390/molecules26123653

**Published:** 2021-06-15

**Authors:** Joselin Aguiar, João L. Gonçalves, Vera L. Alves, José S. Câmara

**Affiliations:** 1 CQM-Centro de Química da Madeira, Universidade da Madeira, Campus Universitário da Penteada, 9020-105 Funchal, Portugal; joselin.aguiar@staff.uma.pt (J.A.); jluis@staff.uma.pt (J.L.G.); vera.alves@staff.uma.pt (V.L.A.); 2 Faculdade de Ciências Exatas e da Engenharia, Universidade da Madeira, Campus da Penteada, 9020-105 Funchal, Portugal

**Keywords:** volatile composition, bioactive properties, HS-SPME/GC-MS

## Abstract

In recent years, there has been a growing interest in studying and exploring the potential health benefits of foods, mainly from vegetables and fruits from regular intake. The presence of secondary metabolites, namely polyphenols, carotenoids and terpenes, in certain food matrices seems to contribute to their functional properties, expressed through an increased prevention in the development of certain chronic diseases, namely coronary heart diseases, neurodegenerative diseases, cancer and diabetes. However, some foods’ volatile secondary metabolites also present important bioactive properties, although this is a poorly scientifically explored field. In this context, and in order to explore the potential bioactivity of volatile metabolites in different vegetables and fruits from regular consumption, the volatile composition was established using a green extraction technique, solid phase microextraction in headspace mode (HS-SPME), combined with gas chromatography tandem mass spectrometry (GC-MS). A total of 320 volatile metabolites, comprising 51 terpenic compounds, 45 organosulfur compounds, 31 aldehydes, 37 esters, 29 ketones, 28 alcohols, 23 furanic compounds, 22 hydrocarbons, 19 benzene compounds, 13 nitrogenous compounds, 9 carboxylic acids, 7 ethers, 4 halogenated compounds and 3 naphthalene derivatives, were positively identified. Each investigated fruit and vegetable showed a specific volatile metabolomic profile. The obtained results revealed that terpenic compounds, to which are associated antimicrobial, antioxidant, and anticancer activities, are the most predominant chemical family in beetroot (61%), orange carrot (58%) and white carrot (61%), while organosulfur compounds (antiviral activity) are dominant in onion, garlic and watercress. Broccoli and spinach are essentially constituted by alcohols and aldehydes (enzyme-inhibition and antimicrobial properties), while fruits from the Solanaceae family are characterized by esters in tamarillo and aldehydes in tomato.

## 1. Introduction

The exponential increase in the world’s population (estimated to reach 9.8 billion by 2050, about 34% higher than today, according to UN estimates), combined with continuous climate changes and consequent decreasing of agricultural areas due to desertification, strengthened by the global crisis caused by COVID-19, constitutes societal and global concerns that challenge the food sector. Humanity depends heavily on plants, both as a food source and as a natural source of bioactive compounds with health-promoting properties. In the last few decades, the global interest in plant-based foods, namely fruits and vegetables, has increased due to the potential ability of bioactive compounds to scavenge free radicals (ROS and RNS) in addition to their antiproliferative, antimicrobial and anti-inflammatory activities [1,2]. Although these properties have been mainly associated with the presence of polyphenols, glucosinolates, phytosterols, and capsaicinoids, several other secondary metabolites including some volatile metabolites (terpenoids, isothiocyanate, allicin, among others) also exert several bioactive functions. Several studies reported the relationship between the dietary intake of high amounts of polyphenol-rich foods and a low incidence of cancer, cardiovascular diseases and inflammation [3,4,5]. These metabolites have an important role in nature since they can function as an indirect plant defense against herbivores and pathogenic microorganisms, but also against extreme abiotic factors such as temperature and sun exposure, humidity, lack of nutrients, among others [6,7,8]. Herbivore-induced VOCs can provide signals for neighboring undamaged plants, acting as messenger compounds that transmit selective information between species to ready their inducible anti-herbivore defenses [9]. In addition, VOCs are generally involved in the attraction of pollinators, and within the complex VOC mixtures emitted by plants, pollinators only detect a part of the compounds and use a portion of them as a signal to find their resource [10].

There are three major groups of secondary metabolites originated by the biosynthesis of primary metabolism: (i) the group of terpenes derived from malonic acid or pyruvate and 3-phosphoglycerate; (ii) the group of nitrogen compounds derived from aromatic amino acids, such as tryptophan and tyrosine and aliphatic amino acids such as ornithine and lysine; and finally (iii) the group of phenolic compounds (derived from shikimic acid or malonic acid) [11,12].

Typically, these compounds are found in fruits and vegetables in small amounts, varying from species to species, and sometimes between varieties in its chemical nature [13]. According to some studies, changes in the content of these compounds can also be influenced by environmental and agronomic conditions, the state of maturity, storage conditions and the time of exposure to UV radiation [14,15,16]. In general, these compounds belong to several chemical families, such as aldehydes, esters of short alkyl chains, terpenes (mainly mono and sesquiterpenes), in addition to nitrogenous and organosulfur compounds [14]. Recently, many of these compounds have become highly recognized due to their biological properties related to health benefits [17]. Terpenic and organosulfur compounds are responsible for the medicinal properties of numerous plants, such as their antihypertensive [18], anti-inflammatory [19], antibacterial [20,21,22] and anticancer activity [23,24,25]. Many of the volatile organic compounds (VOCs) and semi-volatile organic compounds (SVOCs) of fruits and vegetables are generally present in trace amounts (from several mg L^−1^ to a few ng L^−1^, or even less), which means that an effective extraction technique and a sensitive methodology of analysis are required for their adequate characterization [26]. Solid phase microextraction in headspace mode (HS-SPME) combined with gas chromatography–mass spectrometry (GC-MS) is a suitable and widely used analytical approach for metabolomics studies of VOCs due to its undoubted merits, namely short extraction times, the simplicity of operation, the solventless nature of the process, high sensitivity, reproducibility, and robustness [27]. Moreover, SPME is an environmentally friendly approach which satisfies the requirements of green analytical chemistry and chiefly involves the extraction, concentration, and eventually sample introduction in a single step [28]. This technique has become very popular and gained growing acceptance and increasing use in routine laboratories, industrial applications and research, covering subjects from environmental and indoor air quality to food and biological samples, pesticide residues, drugs, and metallic and organometallic species, among others [28]. HS-SPME/GC-qMS is able to detect VOCs that are present at low concentrations in complex matrices such as fruits and vegetables [29,30,31]. The analyte recovery from headspace by a fiber depends on two closely related but distinct equilibria: the first is the matrix/headspace equilibrium responsible for the headspace composition (measured by its distribution coefficient, K2), and the second is the headspace/polymeric fiber coating equilibrium (measured by its distribution coefficient, K1). Therefore, chemometric analysis of GC-MS profiles may be a suitable method for studying volatiles and determining their bioactive compounds [32].

In the present study, we report the use of HS-SPME/GC-qMS as a reliable methodology for the establishment of the volatile composition of fruits (tomato and tamarillo) and vegetables (beetroot, red and yellow onion, white and orange carrot, garlic, watercress, broccoli and spinach) from regular intake, as a useful strategy to identify volatile metabolites with potential benefit effects on human health. Although these effects are mainly attributed to other types of secondary metabolites—polyphenols, carotenoids, phytosterols, glucosinolates, among others—many volatile compounds are associated to health benefits according to reported in several studies [33,34].

## 2. Results and Discussion

The typical aroma of fruits and vegetables depends on a large number of volatiles and the synergic effects, the chemical nature, and its relative amount and odor threshold, which can be related to the fruit or vegetable composition as well as to ripening and processing conditions. Figure S1 (Supplementary Materials) shows the typical volatile pattern of the investigated fruits and vegetables, obtained by HS-SPME_DVB/CAR/PDMS/_GC-qMS.

A total of 320 volatile metabolites, including 51 terpenic compounds, 45 organosulfur compounds, 31 aldehydes, 37 esters, 29 ketones, 28 alcohols, 23 furanic compounds, 22 hydrocarbons, 19 benzene compounds, 13 nitrogenous compounds, 9 carboxylic acids, 7 ethers, 4 halogenated compounds and 3 naphthalene derivatives, were identified. Table S1 (Supplementary Materials) details the volatile composition of each of the samples analyzed, including retention times, Kovats indices, chemical families, molecular formula, and average areas obtained for each volatile compound.

### 2.1. Volatile Composition of Investigated Fruits and Vegetables

#### 2.1.1. Beetroot

Beetroot (*Beta vulgaris* L.) belongs to the Beta genus and to the Amaranthaceae family. A total of 61 volatile compounds were identified, being mainly characterized by terpenic compounds (61.0%), followed by furanic compounds (20.6%), carboxylic acids (5.6%) and benzene derivatives (5.2%). The principal volatile constituents of beetroot include terpinolene (89), γ-terpinene (75) and 5-hydroxymethylfurfural (261), representing 65.6% of the total volatile profile. In lower amounts are 1-methyl-2-(1-methylethyl)-benzene (85; 3.9%), 5-methylfurfural (197; 3.4%), β-pinene (40; 2.9%) and α-pinene (25; 1.7%). It is noteworthy that among the volatile compounds, it was possible to identify geosmin (267), one of the main characteristic compounds of the beetroot aroma. This sesquiterpene is often considered as an off flavor, being responsible for an earthy odor and flavor [35]. Koubaier et al. [36] reported the effect of α-terpinolene against breast and cervical cancer cell lines. Direct virus inhibition of HSV-1 was reported for several monoterpenes including α-terpinene, γ-terpinene and α-pinene, all identified in beetroot [37].

#### 2.1.2. Carrot

**Figure 1:**
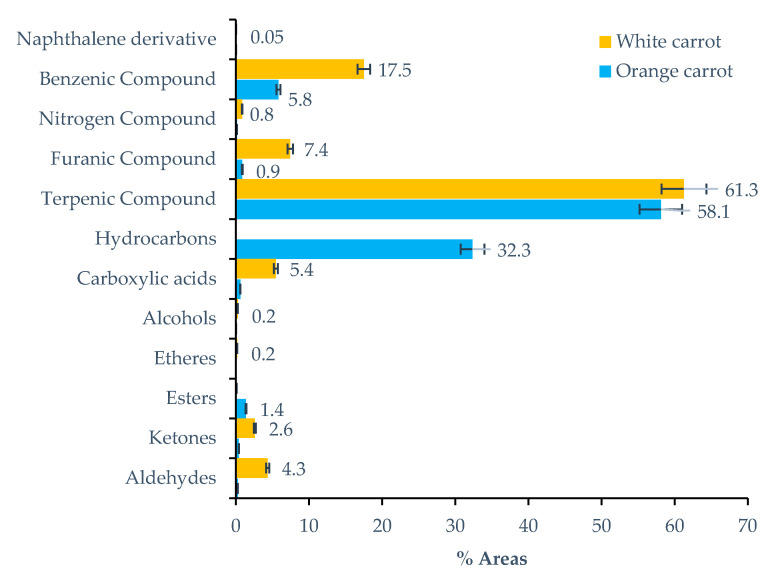
Distribution of chemical families identified in orange and white carrot.

Regarding carrot varieties, 71 volatile metabolites were identified in white carrots, whilst in orange carrots, 59 were identified (Figure 1). The volatile profile of both carrot varieties is mainly composed of terpenic compounds. Orange carrot is very rich in terpenic compounds with 31 being identified, which account for 58.1% of the total volatile fraction of this variety. In white carrot, terpenics are less dominant with 22 volatile metabolites being identified, which represent 61.3% of the total volatile fraction.

The characteristic flavor of fresh carrots is attributed essentially to mono- and sesquiterpenes. Monoterpenes are undoubtedly the most abundant in both varieties, with terpinolene (89) and γ-terpinene (75) being the major volatiles found in white carrot (32.0%) and orange carrot (27.2%), respectively. Manach et al. [38] demonstrated that incubating plasma with γ-terpinene extends the resulting lag phase in the copper-mediated formation of conjugated dienes in LDL by the factor 1.5 or 2.7, respectively. These results agree with those obtained by Graßmann et al. [39], who observed that γ-terpinene can be enriched in LDL by pre-incubating human blood plasma with γ-terpinene. In addition, the same researchers proved that the subsequently isolated LDL shows a high resistance against copper-induced oxidation [39]. α-Pinene (25) was also identified in both varieties, with percentages of around 4.3% in the orange variety and 1.6% in the white variety. According to Güler et al. [40], this compound is primarily responsible for the antimicrobial and antifungal properties of carrots. In addition to these bioactive compounds, D-limonene (59), β-myrcene (51), β-pinene (40) and α-phellandrene (50) were also identified in low quantities.

#### 2.1.3. Onion

**Figure 2:**
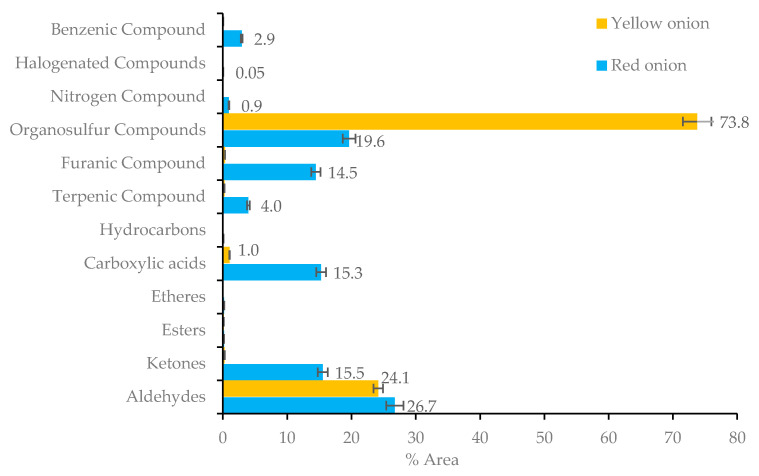
Distribution of chemicals identified in red and yellow onions.

The volatile profile of both (red and yellow) onion varieties is very distinct. Red onions are characterized by several chemical groups, namely aldehydes (26.7%), organosulfur compounds (19.6%) and carboxylic acids (15.3%), while yellow onions are characterized essentially by organosulfur compounds, which account for 73.8% of its total volatile fraction (Figure 2).

The characteristic flavor and the bioactive properties of onions have been mainly attributed to the organosulfur volatile compounds [41], which are biosynthesized essentially after the rupture of the cell structure, when the precursor compounds, S-alk(en)yl cysteine sulfoxides, come into contact with the alliinase enzyme to produce alkenyl sulfenic acids [42]. Many of these compounds have been shown to have antidiabetic and insulinotropic activity in in vivo animal studies [43]. Some studies have demonstrated that the antimicrobial activity of sulfides is affected by the number of carbon atoms of the alk(en)yl groups and sulfur atoms and by the concentration of the diverse sulfur compounds in the volatile fraction of onion, thus emphasizing the importance of characterizing the volatile profile of the onion in the different forms used [41,44,45].

Among the main organosulfur compounds identified in the studied onion varieties, dipropyl disulfide (47.2%, yellow onion), dimethyl trisulfide (8.8% red onion, and 9.4% yellow onion) and dipropyl trisulfide (4.3%, yellow onion) are predominant.

Another class of volatile compounds commonly detected in fresh onion is volatile aldehydes [41], with 2-methyl-2-pentenal (55) produced by the sequential transformation of 1-propenylsulfenic acid to thiopropanal S-oxide, which subsequently originates 2-methyl-2-pentenal [46], the major compound of this family (22.3% in red onion and 9.8% in yellow onion).

#### 2.1.4. Garlic

Like onions, garlic is rich in organosulfur compounds that are responsible for the characteristic odor and taste of this vegetable, as well as bioactive properties [47,48]. Of the 21 compounds identified in the garlic samples, 99.7% of the volatile profile is constituted by organosulfur compounds, with the remaining 0.3% belonging to the esters, ethers, aldehydes and benzene derivatives.

**Figure 3:**
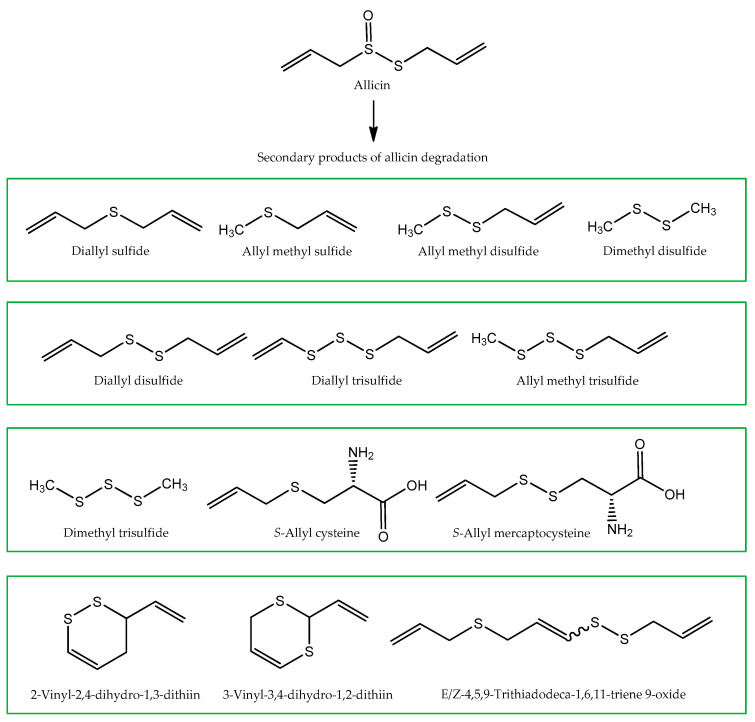
Chemical structures of the main by-products of the degradation of allicin present in garlic.

Diallyl disulfide (175) is the major compound found in the investigated garlic samples, contributing to 70.4% of the total volatile composition. This is one of the most important organosulfur compounds present in garlic, being formed by the decomposition of allicin, an unstable and highly reactive molecule. It is the most common bioactive compound present in garlic and accounts for about 70% of the sulfur compounds family [49]. Allicin is produced enzymatically by the interaction of the non-protein amino acid alliin with the enzyme alliinase, common in all Allium species [50]. During the enzymatic reaction, ammonia and pyruvate are also formed. Due to its instability, allicin degrades easily over time, forming a collection of allyl sulfates and second generation polysulfides (commonly called garlic organosulfides) [51,52]. The chemical structures of the main second generation organosulfides are shown in Figure 3.

Diallyl trisulfide (260) was also identified in a considerable percentage (12.4%), being the second most dominant volatile in garlic. Both diallyl disulfide and diallyl trisulfide, which are associated with anticancer properties [53,54,55], are partly responsible for the bioactive effects of garlic.

#### 2.1.5. Broccoli

It was possible to identify a total of 88 volatile metabolites in broccoli samples, of which 59% were alcohols and 26% aldehydes, the most representative chemical classes. The volatiles with the greatest influence on the volatile composition of the broccoli are (*Z*)-3-hexen-1-ol (136), hexanal (38), heptanal (62), nonanal (133) and pentanal (21) [56]. (*Z*)-3-Hexen-1-ol and hexanal contribute with 43.1% and 10.3%, respectively, for the total volatile composition. Broccoli is also characterized by organosulfur compounds, namely dimethyl disulfide (36) and methyl thiocyanate (99) formed from glucosinolates and amino acid precursors [57,58]. As described by Kebede et al. [58], the main reactions responsible for the formation of VOCs in broccoli can be attributed to (i) the degradation of unsaturated fatty acids; (ii) enzymatic hydrolysis of sulfur-containing amino acids and glucosinolates resulting from the loss of cellular integrity; (iii) thermally induced glucosinolate and the degradation of sulfur-containing amino acids; and finally (iv) the Maillard reaction and its successive side reactions.

#### 2.1.6. Watercress

**Figure 4:**
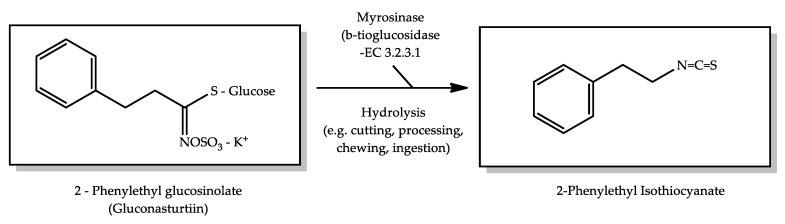
Formation of the 2-phenylethyl isothiocyanate compound through the synthesis of 2-phenylethyl glucosinolate.

According to Kebede et al. [58], the main reactions responsible for the formation of VOCs in broccoli can be attributed to (i) the degradation of unsaturated fatty acids; (ii) enzymatic hydrolyses of sulfur-containing amino acids due to the loss of cellular integrity; (iii) thermal induction degradation of the sulfur-containing amino acids; and (iv) Maillard and Strecker reactions. Glucosinolates are characteristic compounds of the Brassicaceae family, such as watercress and broccoli. Although these compounds are not biologically active, their enzymatic derivatives such as isothiocyanates are responsible for the bioactive properties of watercress [59,60]. 2-Phenylethyl isothiocyanate (309), derived from the synthesis of gluconasturtiin (Figure 4), was found mainly in watercress, contributing with 96.5% to the total volatile profile. It has been associated with several biological properties, including anticancer, antioxidant and antimicrobial properties [60,61].

#### 2.1.7. Spinach

Regarding the characterization of the volatile profile of spinach, 57 volatile metabolites were identified, mainly 14 esters, 13 terpenic compounds, 8 alcohols and 7 aldehydes. Higher alcohols are the most dominant chemical group, accounting for 43.5% of the total volatile fraction, followed by esters (25.6%), aldehydes (16.5%) and terpenic compounds (10.9%).

**Figure 5:**
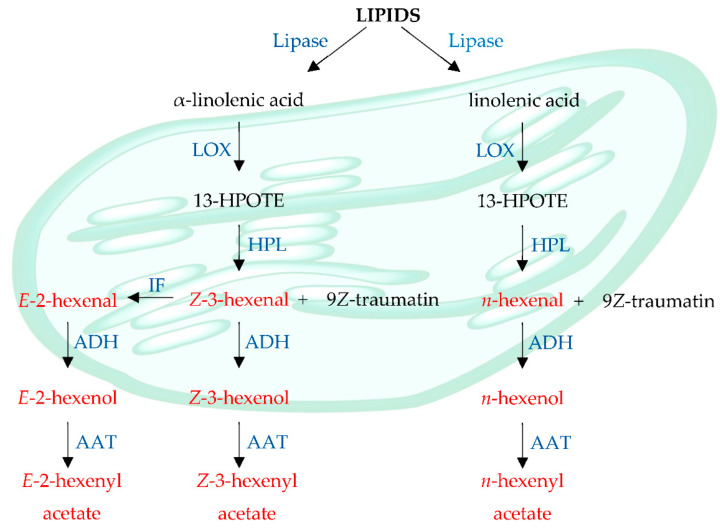
Scheme of the biosynthesis of volatile compounds via lipoxygenase.

C6 alcohols and aldehydes, with an odor similar to cut grass, are typical compounds produced by green leafy vegetables, such as spinach and esters [62]. The major compounds identified in this vegetable were (*E*)-2-hexen-1-ol (148), 2-hexenal (76) and 1-hexanol (126), which together account for 56.8% of the total volatile fraction. These volatile compounds are derived from C18 unsaturated fatty acids such as linoleic acid and/or α-linolenic via the lipoxygenase/hydroperoxide lyase pathways [63]. Once deoxygenated by lipoxygenases (LOX), the resulting hydroperoxide fatty acids are metabolized by several enzymes, including hydroperoxide lyase (HPL), to produce volatile compounds (Figure 5).

C6 aldehydes responsible for the ‘herbaceous’ aroma are formed from 13-hydroperoxides and include hexanal and (*Z*)-3-hexenal. The latter is an unstable compound and is rapidly isomerized to (*E*)-2-hexenal by (3*Z*):(2*E*)-enal isomerase [62]. These aldehydes, in turn, can be transformed into the corresponding alcohols and esters through the activity of alcohol dehydrogenase (ADH) and alcohol acyltransferase (AAT). According to Deng et al. [64], these volatile compounds have bacterial antiproliferative activity, and unsaturated (*E*)-2-hexenal and (*E*)-2-hexen-1-ol volatiles have higher activity than hexanal saturated volatiles and 1-hexanol.

#### 2.1.8. Tomato

Tomato (*Lycopersicon esculentum* L.) is rich in folate, vitamin C, and potassium. Carotenoids, namely lycopene, the most prominent carotenoid, followed by β-carotene, γ-carotene and phytoene, as well as several minor carotenoids, are the most abundant phytonutrients, in addition to a wide variety of volatile compounds. Of the 77 identified volatiles, aldehydes and furanic compounds constitute the most predominant classes, representing about 71% of the total volatile profile. The remaining identified groups contributing to the aroma and taste of this sample were alcohols (6.6%), esters (5.9%), terpenic compounds (5.5%), carboxylic acids (4.7%), organosulfur compounds (2.9%) and ketones (1.6%). The remaining chemical families identified contribute less than 1% to the total volatile profile of tomato.

The individual compounds identified in this study, hexanal (38), (*Z*)-3-hexenal (49), 3-methyl-butanal (11), (*E*)-3-hexen-1-ol (128), β-ionone (289) and 2-isobutylthiazole (138), are considered the most important volatile components for defining the aroma of tomatoes, giving a fresh character to this matrix [28]. The main precursors of these volatile compounds in tomatoes are free amino acids, fatty acids and carotenoids [28,65].

#### 2.1.9. Tamarillo

Regarding the volatile composition of tamarillo (Solanum betaceum), it was possible to identify a total of 65 volatile metabolites, of which 20 were terpenic compounds, 17 esters, 7 alcohols, 5 benzene compounds, 4 aldehydes, 4 furan compounds and the remaining 7 miscellaneous compounds. Ethyl esters were the most important family (65.4%), followed by terpenic compounds (15.8%). Considering the individual metabolites (Table S1), ethyl butyrate (30), methyl hexanoate (64) and ethyl hexanoate (77) are found to be the main identified esters, which together represent 58.6% of the total volatile profile. In lower amounts are eucalyptol (2.9%), D-limonene (1.3%), α- and β-pinene (≤0.6%), which are associated with bioactive effects including anti-inflammatory, antioxidant, antimicrobial and anticancer properties [66].

### 2.2. Potential Bioactive Properties of Volatile Metabolites Present in Studied Vegetables and Fruits

The biological activities and role of many volatile metabolites, so far identified, are partly known. Many of these compounds have proven to play a key role in the prevention and treatment of several diseases such as cancer [67], inflammatory diseases [68], diabetes [69], cardiovascular diseases [70,71], among others.

Among these metabolites, terpenic compounds are one of the most dominant chemical classes found in fruits and vegetables, with several biological effects including antimicrobial, antifungal, antiviral, anti-hyperglycemic, anti-inflammatory, and antiparasitic effects [72]. Table 1 shows some identified volatile metabolites identified in the investigated samples and the corresponding biological effects.

An interesting study about the potential antioxidant activity of monoterpene and sesquiterpene compounds demonstrated that monoterpene hydrocarbons, those with strongly activated methylene groups in their structure such as terpinolene, α-terpinene, γ-terpinene and sabinene, were the most active compounds [34].

Other studies have proved the anticancer properties of several terpenoid compounds acting on different stages of tumor development, such as inhibition of the early initiation and progression of tumorigenesis by inducing cell cycle arrest, tumor cell differentiation, and apoptosis, and in the late stages, the suppression of angiogenesis, invasion, and metastasis through the regulation of various intracellular signaling pathways [98].

Another chemical family, identified in the investigated samples, with interesting biological properties is organosulfur compounds. These compounds are highly reactive phytochemical metabolites, usually found in cruciferous vegetables such as broccoli, cauliflower, brussels sprouts, garlic and onions [99]. Organosulfur compounds are derived from the degradation of sulfur-containing amino acids and have been linked with alliaceous, sulfuric, sweaty, onion and cabbage aromas, contributing to the characteristic flavor of Alliums [41]. Some studies have demonstrated that organosulfur compounds showed several biological properties, including antihyperlipidemic properties [100,101], hypoglycemic actions [102], and anti-inflammatory [103,104], antibacterial [105] and anticancer activity [52]. Diallyl disulfide from garlic and its metabolite allyl mercaptan have been shown to increase histone H3 acetylation in HT-29 and Caco2 cell lines, playing a role in its protective properties on colon carcinogenesis [106]. As demonstrated by Druesne et al. [106], in HT-29 cells and Caco-2, diallyl disulfide could inhibit cell proliferation through the inhibition of histone deacetylase (HDAC) activity, histone hyperacetylation and an increase in p21(waf1/cip1) expression.

## 3. Materials and Methods

### 3.1. Reagents and Materials

All reagents used were analytical quality. Sodium chloride (NaCl, 99.5%) used to obtain the adequate ionic strength was supplied by Panreac (Barcelona, Spain). Ultrapure water (18 MΩ cm at 23 °C) was obtained from a Milli-Q system (Millipore, Bedford, MA, USA). The glass vials, SPME fiber coated with divinylbenzene/carboxen on polydimethylsiloxane (DVB/CAR/PDMS) and SPME holder for manual sampling were purchased from Supelco (Bellefonte, PA, USA). The retention index (RI) was calculated through injection of a series of C8 to C20 straight-chain n-alkanes (concentration of 40 mg L^−1^ in n-hexane) supplied from Fluka (Buchs, Switzerland).

### 3.2. Samples

Triplicates (1 kg) of different vegetables from regular consumption, namely tomato (Solanum lycopersicum), tamarillo (Solanum betaceum), watercress (Rorippa nasturtium-aquaticum), broccoli (Brassica oleracea), spinach (Spinacia oleracea), white carrot and orange carrot (Daucus carota), beetroot (Beta vulgaris), yellow onion and red onion (Allium cepa), and garlic (Allium sativum), were analyzed. The samples were purchased in a commercial area at Funchal, Madeira Island, Portugal. Vegetables with unpleasant appearance were discarded. Each sample was washed, and all inedible parts removed. After trituration to obtain the pulps, the samples were stored at −20 °C in the dark until analysis.

### 3.3. HS-SPME Procedure

The application of an effective HS-SPME method generally requires the optimization of the main experimental factors that influence the extraction process and the experimental response. HS-SPME was performed based on the conditions described by [28] and Perestrelo et al. [26], with slight modifications. For headspace sampling, 4 g of fruit or vegetable pulp, 0.5 g of NaCl, 5 mL of ultra-pure Milli-Q water and a magnetic stirrer were added into a 20 mL amber glass vial. Then, the vial was capped with a PTFE-faced silicone septum and placed in a thermostatic bath at 40 ± 1 °C with constant magnetic stirring (800 rpm).

The SPME fiber (DVB/CAR/PDMS) was exposed to the headspace for 45 min. After extraction, the fiber was withdrawn into the holder needle, removed from the vial and immediately introduced into GC injector port for 6 min at 250 °C for thermal desorption of the VOCs and SVOCs. All analyses were carried out in triplicate (*n* = 3). It is important to note that the fiber was conditioned according to the manufacturer’s instructions before use and before the first daily extraction.

### 3.4. GC-MS Conditions

Chromatographic separations of VOCs and SVOCs from fruits and vegetables were performed using an Agilent Technologies 6890N (Palo Alto, CA, USA) gas chromatography system equipped with a BP-20 fused silica capillary column (30 m × 0.25 mm i.d. × 0.25 μm film thickness) supplied by SGE (Darmstadt, Germany) with helium (Helium N60, Air Liquid, Portugal) as carrier gas at a flow rate of 1 mL × min^−1^ (column-head pressure: 13 psi). The injector temperature was fixed at 250 °C and a splitless injector equipped with an insert of 0.75 mm i.d. was used. The temperature program was set up as follows: initial temperature 40 °C for 1 min, 1.2 °C × min^–1^ ramp until 80 °C, held for 2 min, 3 °C × min^–1^ ramp until 150 °C, held for 2 min, 40 °C × min^–1^ ramp until 220 °C and then held isothermally at 220 °C for 10 min. MS detection was performed in full scan in an Agilent 5975 quadrupole inert mass selective detector, the ion energy used for the electron impact (EI) was 70 eV and the source temperature was 230 °C. The electron multiplier was set to the autotune procedure. The mass acquisition range, made in full scan mode, was 30–300 *m*/*z*.

Volatile metabolite identification was achieved by comparing the retention times (RT) of the chromatographic peaks with those, when available, of authentic standards (ST) run under the same conditions. For metabolites whose reference substances were not available, the identification was performed by matching their retention indices (RI) determined relative to the retention time of a series of n-alkanes (C8–C20) with linear interpolation, with those of literature data, and by comparing their mass spectra using the National Institute of Standards and Technology (NIST) MS 05 spectral database (Gaithersburg, MD, USA).

## 4. Conclusions

The characterization of the volatile composition of fruits and vegetables from regular consumption was carried out using the HS-SPME/GC-qMS methodology. A total of 320 volatile compounds, belonging to different chemical families, were identified. According to the results obtained, it was found that terpenic compounds were predominant in beetroot and in both carrot varieties (orange and white), whereas organosulfur compounds were found almost exclusively in garlic, watercress and in both onion varieties (red and yellow). These chemical compounds are responsible for the bioactive properties, such as antihypertensive, anti-inflammatory, antibacterial, and anticancer properties, among others, of numerous plants.

The data presented here showed the potential of a solvent-free and high-throughput extraction technique to establish the volatile metabolomic pattern of different fruits and vegetables, allowing the establishment of its relationship with their bioactive activities. The knowledge of volatile composition is a valuable tool to improve the aroma quality and the nutritional value of the fruits and vegetables, as well as to identify which metabolites are biologically the most important and can be used for specific purposes as nutraceuticals. The study intends to draw attention to the importance of volatile compounds that constitute fruits and vegetables in human health. Although the vast majority of work on this thematic is directed to polyphenolic compounds, flavonoids and non-flavonoids, powerful bioactive effects are also attributed to volatile compounds, with a great impact on the prevention of certain pathologies such as cancer and cardiovascular and neurodegenerative diseases.

## Figures and Tables

**Table 1:** Biological effects of some volatile metabolites identified in the studied fruits and vegetables.

Volatile Metabolites	Biological Effects	Sample	Reference
β-Myrcene	Analgesic, anti-inflammatory, antibiotic, anticancer, antioxidant	Beetroot, orange carrot, spinach,	[73,74]
Camphene	Antimicrobial, antioxidant	Beetroot, red onion, orange and white carrot, spinach	[75,76]
α-Terpinene	Antimicrobial	Beetroot, orange and white carrot, tomato, tamarillo	[77,78]
Terpinolene	Antioxidant	Beetroot, red onion, orange and white carrot	[79]
γ-terpinene	Anti-inflammatory, antioxidant	Beetroot, red and yellow onion, orange and white carrot, tomato, tamarillo	[39,80]
β-Phellandrene	Antibacterial, anticancer	Beetroot, orange and white carrot	[81,82]
β-Pinene	Antitumor, anti-inflammatory, antimicrobial, antioxidant, antineoplastic, chemoprotective	Beetroot, orange and white carrot, spinach, tamarillo	[80,83,84]
D-Limonene	Antimutagenic, antitumor, antioxidant, antimicrobial, antiproliferative, chemoprotective	Beetroot, orange and white carrot, yellow onion, broccoli, spinach, tomato, tamarillo	[80,84,85]
Eucalyptol	Anti-inflammatory	Broccoli, tomato	[86]
Geraniol	Chemopreventive activity, antimutagenic, anti-inflammatory	Tomato	[86,87]
Eugenol	Anticancer, antimicrobial, antioxidant	Tomato, tamarillo	[79]
β-ionone	Chemopreventive activity	Orange carrot, broccoli, watercress, spinach, tomato, tamarillo	[87]
α-Pinene	Antimicrobial, anticancer, anti-inflammatory, antiallergic	Beetroot, red onion, orange and white carrot, broccoli, watercress, spinach, tomato, tamarillo	[88,89]
3-Carene	Antimicrobial, antioxidant, anticancer,	Orange and white carrot, spinach, tamarillo	[90],
Diallyl disulfide	Anticancer, antioxidant, anti-inflammatory	Garlic	[91,92]
Diallyl trisulfide	Anticancer, antidiabetic	Garlic	[93,94]
Dipropyl disulfide	Anticancer	Yellow onion	[95]
Dimethyl trisulfide	Reduction in acute pancreatitis	Yellow and red onion	[96]
2-Phenylethyl glucosinolate	Chemopreventive	Watercress	[97]

## Supplementary Materials

The following are available online, Figure S1: Typical HS-SPMEDVD/CAR/PDMS/GC-qMS chromatograms of the volatile composition of investigated fruits and vegetables, Table S1: Volatile composition identified in eleven fruits and vegetables using HS-SPMEDVB/CAR/PDMS/GC-MS methodology.
