# Supplementary material for: Relationship between Volatile Composition and Bioactive Potential of Vegetables and Fruits of Regular Consumption—An Integrative Approach
Source: Molecules. 2021 Jun 15;26(12):3653. doi: 10.3390/molecules26123653 (PMC8232647; doi:10.3390/molecules26123653)
Supplement: Supplementary file 1 [file molecules-26-03653-s001.zip › molecules-1241631-supplementary.pdf]

Relationship between Volatile Composition and Bioactive Potential of Vegetables and Fruits of Regular Consumption—An Integrative Approach

Joselin Aguiar [1], João L. Gonçalves[1], Vera L. Alves[1], José S. Câmara [1,2,*]

[1] CQM - Centro de Química da Madeira, Universidade da Madeira, Campus Universitário da Penteada, 9020-105 Funchal, Portugal
[2] Faculdade de Ciências Exatas e da Engenharia, Universidade da Madeira. Campus da Penteada, 9020-105 Funchal, Portugal.
* Correspondence: jsc@staff.uma.pt; Tel.: +351 291705112; Fax: +351 291705149

Supporting Information

Table of Contents

Figure S1. Chromatograms of the volatile fraction of the 11 fruit and vegetable samples obtained by HS_SPMGC-qMS.

Table S1. Volatile composition identified in eleven fruits and vegetables using HS-SPMEDVB/CAR/PDMS/GC-MS methodology.

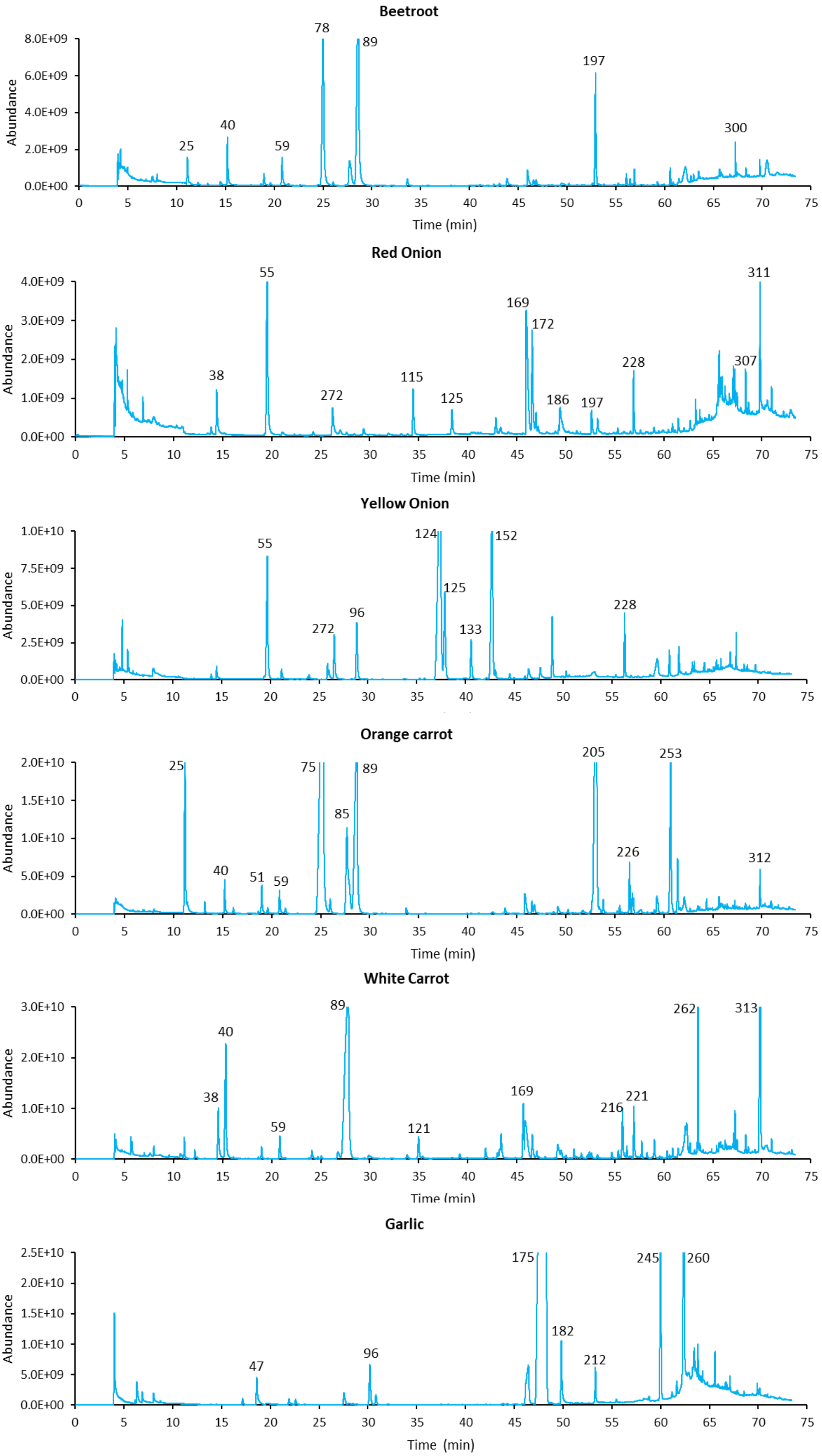
Beetroot
Abundance
Time (min)
78
89
197
40
25
59
300
Red Onion
Abundance
Time (min)
55
311
169
172
38
272
115
125
228
307
186
197
Yellow Onion
Abundance
55
124
152
125
96
272
133
228
Orange carrot
Abundance
Time (min)
25
75
89
85
40
51
59
205
253
226
312
White Carrot
Abundance
89
40
38
59
121
169
216
221
262
313
Garlic
Abundance
Time (min)
175
245
260
182
212
47
96

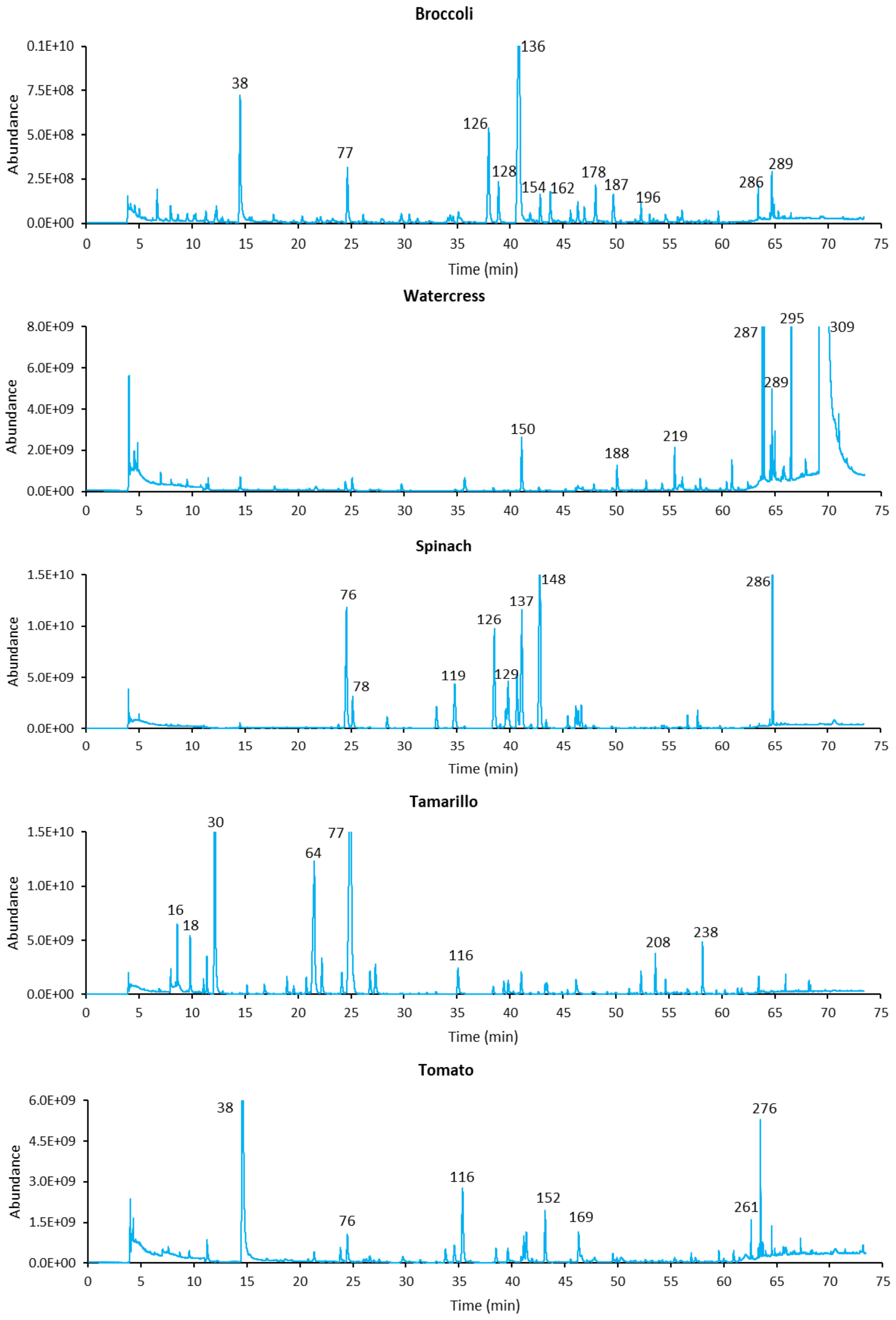

Figure S1. Chromatograms of the volatile fraction of the 11 fruit and vegetable samples obtained by HS_SPMGC-qMS.

Table S1. Volatile composition identified in eleven fruits and vegetables using HS-SPMEDVB/CAR/PDMS/GC-MS methodology.

Peak No.	RT[a] (min)	RI_{calc}[b]	Volatile metabolite	Chemical class	MF[c]	Total peak area ($\times 10^6 \pm \sigma$)										
						Beetroot	Red onion	Yellow onion	Orange carrot	White carrot	Garlic	Broccoli	Watercress	Spinach	Tomato	Tamarillo
1	4.628	1065	Acetaldehyde	Aldehydes	C_2H_4O							0.81 ± 0.13				
2	4.972	1073	Dimethyl sulfide	Organosulfur compounds	C_2H_6S									0.87 ± 0.06		
3	5.033	1074	4-Methyl-heptane	Hydrocarbons	C_8H_{18}							0.84 ± 0.24				
4	5.359	1081	Propanal	Aldehydes	C_3H_6O		3.69 ± 0.14					0.26 ± 0.03				
5	5.583	1085	Acetone	Ketones	C_3H_6O					4.44 ± 0.09						
6	6.548	1105	Aminomethanesulfonic acid	Organosulfur compounds	CH_5NO_3S						15.8 ± 2.90					
7	6.696	1110	2,4-Dimethyl-1-heptene	Hydrocarbons	C_9H_{18}							3.28 ± 0.10				
8	6.803	1113	Ethyl acetate	Esters	$C_4H_8O_2$					0.48 ± 0.04		0.30 ± 0.21				1.40 ± 0.29
9	6.944	1118	Allyl mercaptan	Organosulfur compounds	C_3H_6S						9.40 ± 2.05					
10	7.416	1132	2-Methyl-Butanal	Aldehydes	$C_5H_{10}O$							0.36 ± 0.15				
11	7.469	1133	3-Methyl-Butanal	Aldehydes	$C_5H_{10}O$	0.87 ± 0.04						0.59 ± 0.10		0.28 ± 0.02	0.73 ± 0.11	
12	7.971	1147	Ethyl aminomethylformimidate	Nitrogen compounds	$C_4H_{10}N_2O$		1.47 ± 0.44									
13	7.977	1147	Ethyl alcohol	Alcohols	C_2H_6O				1.04 ± 0.45	5.43 ± 0.46		5.58 ± 0.36	0.79 ± 0.05	0.75 ± 0.1		8.65 ± 0.99
14	8.008	1148	Ethanediamide	Nitrogen compounds	$C_2H_4N_2O_2$	1.13 ± 0.38										
15	8.081	1150	Methylthiirane	Organosulfur compounds	C_3H_6S			2.17 ± 0.85			8.79 ± 1.59					
16	8.611	1163	(Z)-3-methyl-1,3,5-hexatriene	Hydrocarbons	C_7H_{10}											29.0 ± 0.28
17	8.622	1164	2-Ethylfuran	Furanic compounds	C_6H_8O							1.57 ± 0.71			0.62 ± 0.10	
18	9.827	1192	Methyl butanoate	Esters	$C_5H_{10}O_2$											23.5 ± 0.99
19	9.1	1175	2-Propenyl-cyclopentane	Hydrocarbons	C_8H_{14}								0.27 ± 0.13			
20	9.579	1186	3-Pentanone	Ketones	$C_5H_{10}O$								1.60 ± 0.14			
21	9.498	1184	Pentanal	Aldehydes	$C_5H_{10}O$					1.35 ± 0.50		3.13 ± 0.34			1.17 ± 0.20	
22	10.139	1198	2,6-Dimethylnonane	Hydrocarbons	$C_{11}H_{24}$							0.48 ± 0.20				
23	10.247	1201	Decene	Hydrocarbons	$C_{10}H_{22}$							0.02 ± 0.0003				
24	10.771	1213	3-Ethyl-1,5-octadiene	Hydrocarbons	$C_{10}H_{18}$								0.42 ± 0.11			

25	11.104	1221	α-Pinene	Terpenic compounds	$C_{10}H_{16}$	7.90 ± 1.57	2.42 ± 0.61		166.3 ± 22.4	43.6 ± 1.33		0.17 ± 0.01	0.30 ± 0.05	2.13 ± 0.30	0.10 ± 0.01	8.53 ± 0.28
26	11.247	1224	Methyl isovalerate	Esters	$C_6H_{12}O_2$										2.25 ± 0.30	0.93 ± 0.14
27	11.251	1224	1-Penten-3-one	Ketones	C_5H_8O							3.44 ± 0.28	1.37 ± 0.16			
28	11.375	1227	Trichloromethane	Halogenated compounds	$CHCl_3$	0.23 ± 0.02	0.18 ± 0.04									
29	11.406	1228	α-Thujene	Terpenic compounds	$C_{10}H_{16}$				2.81 ± 0.60	0.71 ± 0.02						
30	12.077	1242	Ethyl butyrate	Esters	$C_6H_{12}O_2$		0.64 ± 0.05									200.7 ± 1.00
31	12.194	1244	Toluene	Benzene compounds	C_7H_8	0.72 ± 0.10		0.32 ± 0.005		7.39 ± 0.32		1.71 ± 0.52			0.16 ± 0.03	
32	12.278	1246	β-2-Butenal	Aldehydes	C_4H_6O							4.35 ± 0.67				
33	12.828	1257	Ethyl 2-methylbutanoate	Esters	$C_7H_{14}O_2$							1.86 ± 0.57				
34	12.896	1258	Methylthiocyclopentane	Organosulfur compounds	$C_6H_{12}S$								0.11 ± 0.01			
35	13.192	1264	Camphene	Terpenic compounds	$C_{10}H_{16}$	0.32 ± 0.07	0.13 ± 0.01		9.58 ± 1.82	1.59 ± 0.26				0.23 ± 0.07		
36	13.931	1278	Dimethyl disulfide	Organosulfur compounds	$C_2H_6S_2$		1.99 ± 0.86	1.27 ± 0.07				0.16 ± 0.06				
37	14.393	1286	4,8-Dimethyl-1,7-nonadiene	Hydrocarbons	$C_{11}H_{20}$								0.32 ± 0.01			
38	14.519	1288	Hexanal	Aldehydes	$C_6H_{12}O$	1.90 ± 0.13	12.8 ± 1.24	4.63 ± 0.96	0.47 ± 0.14	59.4 ± 13.1	0.29 ± 0.13	73.6 ± 12.6		1.50 ± 0.39	119.3 ± 0.40	0.58 ± 0.04
39	15.016	1296	Undecane	Hydrocarbons	$C_{11}H_{24}$				0.39 ± 0.12							
40	15.217	1300	β-Pinene	Terpenic compounds	$C_{10}H_{16}$	13.27 ± 2.38			34.02 ± 6.74	202.8 ± 37.5				0.73 ± 0.30		5.84 ± 0.99
41	15.301	1301	Pyrroliphene	Nitrogen compounds	$C_{23}H_{29}NO_2$		1.10 ± 0.21									
42	16.089	1317	Sabinene	Terpenic compounds	$C_{10}H_{16}$	0.07 ± 0.02										0.23 ± 0.08
43	16.879	1331	2-Methylbutyl acetate	Esters	$C_7H_{14}O_2$										0.55 ± 0.10	
44	16.968	1333	Ethylbenzene	Benzene compounds	C_8H_{10}							0.27 ± 0.07				
45	17.556	1343	3-Carene	Terpenic compounds	$C_{10}H_{16}$				0.57 ± 0.12	0.45 ± 0.20				0.80 ± 0.10		2.65 ± 0.42
46	17.675	1345	(E)- 2-Pentenal	Aldehydes	C_5H_8O							2.47 ± 0.09	2.34 ± 0.005			
47	18.179	1354	Diallyl sulfide	Organosulfur compounds	$C_6H_{10}S$						31.6 ± 5.76					
48	18.379	1357	Pentyloxirane	Ethers	$C_7H_{14}O$							0.32 ± 0.01			0.30 ± 0.04	
49	18.604	1361	(Z)-3-Hexenal	Aldehydes	$C_6H_{10}O$										0.74 ± 0.10	
50	18.666	1362	α-Phellandrene	Terpenic compounds	$C_{10}H_{16}$	0.32 ± 0.05			2.94 ± 1.10	2.16 ± 0.69						0.33 ± 0.04
51	18.963	1367	β-Myrcene	Terpenic compounds	$C_{10}H_{16}$	2.96 ± 0.70			40.7 ± 5.58					0.33 ± 0.03		

52	19.097	1369	β-Thujene	Terpenic compounds	$C_{10}H_{16}$				2.77 ± 0.31	0.88 ± 0.06						7.11 ± 0.85
53	19.612	1377	α-Terpinene	Terpenic compounds	$C_{10}H_{16}$	0.63 ± 0.16			9.27 ± 1.59	1.86 ± 0.21					0.64 ± 0.10	4.90 ± 0.99
54	19.61	1377	4-Methylhexanal	Aldehydes	$C_7H_{14}O$							0.51 ± 0.19				
55	19.661	1378	2-Methyl-2-Pentenal	Aldehydes	$C_6H_{10}O$		72.8 ± 5.92	63.1 ± 1.81							0.29 ± 0.04	
56	20.262	1387	Methyl 3-methyl-2-butenoate	Esters	$C_6H_{10}O_2$											1.07 ± 0.30
57	20.285	1388	1-Penten-3-ol	Alcohols	$C_5H_{10}O$							2.18 ± 0.58	2.01 ± 0.16			
58	20.614	1392	2-Ethyl-thiophene	Organosulfur compounds	C_6H_8S							0.12 ± 0.01				
59	20.793	1395	D-Limonene	Terpenic compounds	$C_{10}H_{16}$	7.36 ± 1.95		0.71 ± 0.43	45.7 ± 2.80	33.4 ± 4.47		2.71 ± 0.001		0.48 ± 0.09	0.74 ± 0.10	11.3 ± 0.99
60	21.019	1398	1,3,4-Thiadiazol-2-amine	Organosulfur compounds	$C_2H_3N_3S$								0.97 ± 0.09			
61	21.165	1401	2,4-Dimethylthiophene	Organosulfur compounds	C_6H_8S		1.24 ± 0.29	4.00 ± 1.05			1.32 ± 0.09					
62	21.233	1402	Heptanal	Aldehydes	$C_7H_{14}O$					1.19 ± 0.30		0.96 ± 0.02				
63	21.328	1404	p-Xylene	Benzene compounds	C_8H_{10}							0.33 ± 0.001				
64	21.346	1404	Methyl hexanoate	Esters	$C_7H_{14}O_2$										6.28 ± 0.30	162.0 ± 2.00
65	21.594	1409	2-Heptanone	Ketones	$C_7H_{14}O$							0.10 ± 0.003				
66	21.473	1407	β-Phellandrene	Terpenic compounds	$C_{10}H_{16}$	0.36 ± 0.16			5.43 ± 1.01	2.08 ± 0.06						
67	22.642	1428	Dodecane	Hydrocarbons	$C_{12}H_{26}$							0.59 ± 0.13				
68	22.999	1435	Eucalyptol	Terpenic compounds	$C_{10}H_{18}O$							0.42 ± 0.19				25.7 ± 0.99
69	23.629	1446	4-Methyl-2-heptanone	Ketones	$C_8H_{16}O$							0.71 ± 0.16				
70	23.803	1449	2-Pentylfuran	Furanic compounds	$C_9H_{14}O$			0.39 ± 0.14				0.39 ± 0.10			3.75 ± 0.59	
71	23.961	1451	Bicyclo[2.2.2]oct-5-en-2-one	Ketones	$C_8H_{10}O$			0.13 ± 0.02								
72	23.966	1451	Methyl propyl disulfide	Organosulfur compounds	$C_4H_{10}S_2$		0.21 ± 0.01	1.14 ± 0.18								
73	24.441	1459	(E)-2-Hexenal	Aldehydes	$C_6H_{10}O$								3.12 ± 0.38			
74	24.223	1456	(E)-Ocimene	Terpenic compounds	$C_{10}H_{16}$	0.46 ± 0.14				0.95 ± 0.81			4.07 ± 0.94	4.81 ± 0.59		
75	24.651	1463	γ-Terpinene	Terpenic compounds	$C_{10}H_{16}$	91.2 ± 13.8	4.18 ± 0.16	0.19 ± 0.02	1040.9 ± 140.7	312.6 ± 24.09					0.27 ± 0.03	24.7 ± 2.97
76	24.653	1463	2-Hexenal	Aldehydes	$C_6H_{10}O$							36.3 ± 13.0		42.0 ± 0.10	20.3 ± 0.31	
77	24.683	1463	Ethyl hexanoate	Esters	$C_8H_{16}O_2$			0.78 ± 0.06		2.74 ± 0.18		4.09 ± 1.61				145.6 ± 0.57
78	25.699	1480	(Z)-Ocimene	Terpenic compounds	$C_{10}H_{16}$	0.95 ± 0.38			21.8 ± 4.40	8.88 ± 1.68				19.3 ± 0.20		

79	25.862	1482	2-Methyl-1-butanol	Alcohols	$C_5H_{12}O$										0.63 ± 0.03	
80	26.088	1486	3-Methyl-1-butanol	Alcohols	$C_5H_{12}O$										0.89 ± 0.10	
81	26.21	1488	3,4-Dimethylthiophene	Organosulfur compounds	C_6H_8S		10.1 ± 2.47	27.4 ± 2.33			17.7 ± 1.81					
82	26.759	1496	1,3,5,7-Cyclooctatetraene	Hydrocarbons	C_8H_8										2.10 ± 0.20	
83	27.073	1501	2-Ethyl-1,4-dimethylbenzene	Benzene compounds	$C_{10}H_{14}$									0.40 ± 0.03		
84	27.202	1503	1-methyl-4-(1-methylethyl)-benzene	Benzene compounds	$C_{10}H_{14}$					52.2 ± 0.54						45.0 ± 1.13
85	27.434	1508	1-Methyl-2-(1-methylethyl)-benzene	Benzene compounds	$C_{10}H_{14}$	18.0 ± 4.51	10.0 ± 1.86		215.7 ± 31.9			0.18 ± 0.04		0.38 ± 0.04		22.5 ± 0.14
86	27.598	1511	(Z)-Methyl 3-hexenoate	Esters	$C_7H_{12}O_2$										2.57 ± 0.60	0.45 ± 0.14
87	27.823	1515	1-Pentanol	Alcohols	$C_5H_{12}O$							0.39 ± 0.04			0.82 ± 0.03	
88	28.021	1518	Styrene	Benzene compounds	C_8H_8							0.50 ± 0.28	0.81 ± 0.13			
89	28.22	1522	Terpinolene	Terpenic compounds	$C_{10}H_{16}$	147.2 ± 19.9	6.86 ± 1.82		517.6 ± 38.1	892.2 ± 44.66						
90	28.357	1524	2-Ethylthiophene	Organosulfur compounds	C_6H_8S						0.63 ± 0.14					
91	28.749	1531	Hexyl acetate	Esters	$C_8H_{16}O_2$									4.66 ± 0.20	0.22 ± 0.10	0.59 ± 0.03
92	29.056	1536	2-Ethoxyethyl acetate	Ethers	$C_6H_{12}O_3$		0.15 ± 0.07									
93	29.14	1537	2-Ethoxyethanol	Ethers	$C_4H_{10}O_2$								0.13 ± 0.03	0.23 ± 0.10	0.11 ± 0.04	
94	29.444	1542	(E,E)-2,4-Hexadiene	Hydrocarbons	C_6H_{10}									0.16 ± 0.03		
95	29.446	1542	Methyl heptanoate	Esters	$C_8H_{16}O_2$											0.13 ± 0.01
96	29.515	1544	1,3-Dithiane	Organosulfur compounds	$C_4H_8S_2$		15.9 ± 1.70	38.3 ± 4.95			8.35 ± 1.66					
97	29.572	1544	(Z)-3-Hexenyl formate	Esters	$C_7H_{17}O_2$							0.33 ± 0.02				
98	29.68	1546	Methyl 2-propenyl disulfide	Organosulfur compounds	$C_4H_8S_2$						36.2 ± 3.93					
99	29.709	1547	Methyl thiocyanate	Organosulfur compounds	C_2H_3NS							3.35 ± 0.15	2.36 ± 0.81			
100	30.29	1556	Octanal	Aldehydes	$C_8H_{16}O$	0.65 ± 0.16				12.9 ± 5.19		0.08 ± 0.05			1.03 ± 0.10	
101	30.654	1562	Methyl (E)-2-hexenoate	Esters	$C_7H_{12}O_2$									0.66 ± 0.49		
102	30.704	1563	cis-2-(2-Pentenyl)furan	Furanic compounds	$C_9H_{12}O$								0.13 ± 0.004		0.22 ± 0.10	
103	31.153	1570	trans-2-(2-Pentenyl)furan	Furanic compounds	$C_9H_{12}O$									0.20 ± 0.11		0.95 ± 0.14
104	31.175	1570	Allyl 2-butenoate	Esters	$C_7H_{10}O_2$							1.60 ± 0.50				
105	31.19	1571	Ethyl-3-hexanoate	Esters	$C_8H_{14}O_2$											1.00 ± 0.14

106	31.674	1578	2-Vinylthiophene	Organosulfur compounds	C_6H_6S						1.73 ± 0.46					
107	31.952	1582	2,2,6-Trimethylcyclohexanone	Ketones	$C_9H_{16}O$								0.09 ± 0.03			
108	32.032	1584	Hexanenitrile	Nitrogen compounds	$C_6H_{11}N$							0.14 ± 0.003				
109	32.161	1586	4-(Diethylamino)-2-butanone	Nitrogen compounds	$C_8H_{17}NO$		0.64 ± 0.15									
110	32.364	1589	1-Octen-3-one	Ketones	$C_8H_{14}O$							0.58 ± 0.25			1.14 ± 0.30	
111	33.086	1600	2,3-Dimethyl-1,3-butadiene	Hydrocarbons	C_6H_{10}								0.10 ± 0.04			
112	33.19	1601	2,3-Dimethyloxirane	Ethers	C_4H_8O		0.25 ± 0.02									
113	33.815	1613	(E)-2-Penten-1-ol	Alcohols	$C_5H_{10}O$							0.21 ± 0.12	0.79 ± 0.09			
114	33.879	1614	(Z)-2-Heptenal	Aldehydes	$C_7H_{12}O$			0.53 ± 0.17		4.04 ± 1.83		2.29 ± 0.24			4.59 ± 0.10	
115	34.002	1617	1-hydroxy-2-Propanone	Ketones	$C_3H_6O_2$	4.86 ± 0.47	11.5 ± 2.52	1.70 ± 0.08	3.23 ± 0.54	22.7 ± 6.07					1.49 ± 0.58	
116	34.013	1617	(Z)-3-Hexen-1-ol acetate	Esters	$C_8H_{14}O_2$							1.86 ± 0.05	0.25 ± 0.01	12.5 ± 0.30	0.36 ± 0.04	0.95 ± 0.14
117	34.683	1629	(Z)-2-Penten-1-ol	Alcohols	$C_7H_{12}O$							2.52 ± 0.01	5.43 ± 0.80			
118	35.024	1635	2,3-Octanedione	Ketones	$C_5H_{10}O$							2.67 ± 0.45				
119	35.103	1637	(E)-2-Hexen-1-ol acetate	Esters	$C_8H_{14}O_2$									12.7 ± 0.30		
120	35.345	1641	Hexyl isobutyrate	Esters	$C_{10}H_{20}O_2$									2.43 ± 0.10		
121	35.561	1645	6-Methyl-5-hepten-2-one	Ketones	$C_8H_{14}O$					1.97 ± 0.60		1.01 ± 0.24				0.21 ± 0.06
122	35.956	1652	2,7-Dimethyl-1-octene	Hydrocarbons	$C_{10}H_{20}$										0.28 ± 0.05	
123	37.581	1680	Allyl isothiocyanate	Organosulfur compounds	C_4H_5NS								0.25 ± 0.003			
124	37.624	1681	Dipropyl disulfide	Organosulfur compounds	$C_6H_{14}S_2$			317.4 ± 15.9								
125	37.952	1686	Dimethyl trisulfide	Organosulfur compounds	$C_2H_6S_3$		30.4 ± 5.20	63.4 ± 8.18								
126	38.241	1691	1-Hexanol	Alcohols	$C_6H_{14}O$					0.94 ± 0.30		59.0 ± 4.9	1.13 ± 0.12	34.0 ± 0.20	3.75 ± 0.30	5.73 ± 1.14
127	38.825	1701	Allo-Ocimene	Terpenic compounds	$C_{10}H_{16}$				0.48 ± 0.12							
128	38.95	1703	(E)-3-Hexen-1-ol	Alcohols	$C_6H_{12}O$							16.2 ± 3.64			8.34 ± 0.31	
129	39.882	1721	(Z)-3-Hexenyl butanoate	Esters	$C_{10}H_{18}O_2$								0.19 ± 0.01	15.3 ± 0.49		
130	39.756	1719	1,3,8-p-Menthatriene	Terpenic compounds	$C_{10}H_{14}$	0.36 ± 0.06			0.24 ± 0.03	4.78 ± 1.80						
131	39.885	1721	Methyl octanoate	Esters	$C_9H_{18}O_2$										4.80 ± 0.11	19.2 ± 0.99
132	39.939	1722	(Z)-3-Hexenyl propanoate	Esters	$C_9H_{16}O_2$									2.56 ± 0.30		
133	40.016	1724	Nonanal	Aldehydes	$C_9H_{18}O$			1.58 ± 0.21							0.79 ± 0.30	

134	40.155	1726	2-Nonanone	Ketones	$C_9H_{18}O$					0.53 ± 0.09						
135	40.539	1734	2,3,5,6-Tetramethyl phenol	Alcohols	$C_{10}H_{14}O$											1.05 ± 0.28
136	41.007	1742	(Z)-3-Hexen-1-ol	Alcohols	$C_6H_{12}O$							246.8 ± 21.7	17.4 ± 1.96	0.40 ± 0.21		14.4 ± 2.97
137	41.299	1748	(E)-2-Hexenyl propanoate	Esters	$C_9H_{16}O_2$									4.47 ± 0.39		
138	41.451	1750	2-Isobutylthiazole	Organosulfur compounds	$C_7H_{11}NS$										8.29 ± 0.31	
139	41.643	1754	(E,E)-2,4-Hexadienal	Aldehydes	C_6H_8O							2.30 ± 0.30		0.17 ± 0.03		
140	41.894	1759	3-Octen-2-one	Ketones	$C_8H_{14}O$					9.05 ± 1.91						
141	41.981	1760	3-Ethyl-2-methyl-1,3-hexadiene	Hydrocarbons	C_9H_{16}										0.21 ± 0.03	
142	42.161	1763	3-(4-Methyl-3-pentenyl)-furan	Furanic compounds	$C_{10}H_{14}O$										0.32 ± 0.10	
143	42.191	1764	3,5-Octadien-2-ol	Alcohols	$C_8H_{14}O$							0.55 ± 0.04				
144	42.324	1766	1,2,3,4-Tetramethyl benzene	Benzene compounds	$C_{10}H_{14}$	0.49 ± 0.05										
145	42.348	1767	Hexyl butanoate	Esters	$C_{10}H_{20}O_2$									0.94 ± 0.59		0.58 ± 0.14
146	42.42	1768	6-Methyl-3,5-heptadiene-2-one	Ketones	$C_8H_{12}O$										0.44 ± 0.07	
147	42.688	1773	(E)-2-Octen-1-ol	Alcohols	$C_8H_{16}O$								0.10 ± 0.01			
148	42.701	1773	(E)-2-Hexen-1-ol	Alcohols	$C_6H_{12}O$									86.4 ± 0.20		1.54 ± 0.57
149	42.774	1774	2,3-Dihydro-2-methyl-benzofuran	Furanic compounds	$C_9H_{10}O$				0.97 ± 0.06							
150	42.839	1775	1-Isothiocyanato-3-methylbutane	Organosulfur compounds	$C_6H_{11}NS$								0.88 ± 0.06			
151	42.859	1776	2-ethyl-1,3-dimethylbenzene	Benzene compounds	$C_{10}H_{14}$	0.63 ± 0.03										
152	43.094	1780	(E)-2-Octenal	Aldehydes	$C_8H_{14}O$		1.54 ± 0.08	91.9 ± 6.65		25.8 ± 3.05					13.4 ± 0.61	
153	43.154	1781	Hexyl 2-methylbutanoate	Esters	$C_{10}H_{18}O_2$									0.81 ± 0.10		
154	43.303	1784	Ethyl octanoate	Esters	$C_{10}H_{20}O_2$			0.17 ± 0.08				0.48 ± 0.12				4.83 ± 1.00
155	43.409	1786	(E)-2-Dodecenal	Aldehydes	$C_{12}H_{22}O$	0.36 ± 0.01										
156	43.27	1783	3-(Methylthio)-quinoline	Organosulfur compounds	$C_{10}H_9NS$	0.02 ± 0.004										
157	43.737	1791	p-Cymenene	Terpenic compounds	$C_{10}H_{12}$	2.93 ± 0.22			8.22 ± 1.09	35.9 ± 4.71			0.16 ± 0.03	0.41 ± 0.10		5.04 ± 0.29
158	43.768	1792	1,2-Dichlorobenzene	Halogenated compounds	$C_6H_4Cl_2$										0.11 ± 0.03	
159	43.952	1795	1-Methoxy-4-methylbenzene	Benzene compounds	$C_8H_{10}O$											0.34 ± 0.08
160	44.105	1798	1-Methoxy-3-methylbenzene	Benzene compounds	$C_8H_{10}O$											0.09 ± 0.01

161	44.233	1800	1,4-Dichlorobenzene	Halogenated compounds	$C_6H_4Cl_2$	0.20 ± 0.04								0.18 ± 0.03		
162	44.285	1801	1,3-Ditertiarybutylbenzene	Benzene compounds	$C_{14}H_{22}$						1.15 ± 0.13	10.2 ± 3.66				
163	44.482	1805	(E,E)-Cosmene	Terpenic compounds	$C_{10}H_{14}$					1.10 ± 0.10				0.24 ± 0.10		
164	44.836	1812	2-Methyl-1-propenylbenzene	Benzene compounds	$C_{10}H_{12}$	0.14 ± 0.01										
165	45.056	1816	Diallyl sulfide	Organosulfur compounds	$C_6H_{10}S$			0.77 ± 0.08								
166	45.21	1819	Hexyl n-valerate	Esters	$C_{11}H_{22}O_2$							0.09 ± 0.01				
167	45.321	1821	4-Isothiocyanato-1-butene	Organosulfur compounds	C_5H_7NS								0.46 ± 0.10			
168	45.543	1826	(Z)-2-Hexenyl butyrate	Esters	$C_{10}H_{18}O_2$									0.13 ± 0.03		1.36 ± 0.28
169	45.983	1834	Acetic acid	Carboxylic acids	$C_2H_4O_2$	11.4 ± 2.90	38.5 ± 1.80	7.02 ± 2.25	18.2 ± 2.11	105.8 ± 18.5			0.78 ± 0.07		11.7 ± 0.40	20.4 ± 0.99
170	45.717	1829	1-Octen-3-ol	Alcohols	$C_8H_{16}O$							2.78 ± 0.18			0.69 ± 0.03	
171	45.906	1833	Heptanol	Alcohols	$C_7H_{16}O$							0.56 ± 0.24				
172	46.565	1845	Furfural	Furanic compounds	$C_5H_4O_2$	3.54 ± 0.94	20.2 ± 3.54	0.98 ± 0.09	6.00 ± 1.86	29.1 ± 2.78					1.88 ± 0.40	
173	46.73	1848	(E)-2-Hexenyl butanoate	Esters	$C_{11}H_{22}O_2$									4.47 ± 1.28		3.01 ± 0.28
174	46.95	1852	(Z)-Hex-3-en-1-yl 2-methylbutanoate	Esters	$C_{11}H_{20}O_2$							5.09 ± 0.64		5.31 ± 0.59		
175	47.578	1864	Diallyl disulfide	Organosulfur compounds	$C_6H_{10}S_2$						1217.7 ± 78.67					
176	47.87	1869	4-Ethyl-cyclohexene	Hydrocarbons	C_8H_{14}										2.83 ± 0.13	
177	47.947	1871	(E,E)-2,4-Heptadienal	Aldehydes	$C_7H_{10}O$					2.14 ± 0.78		6.31 ± 2.14		1.04 ± 0.49		
178	47.996	1872	(Z)-3-Hexenyl pentanoate	Esters	$C_{11}H_{20}O_2$							8.63 ± 0.11				
179	48.286	1877	Decanal	Aldehydes	$C_{10}H_{20}O$	0.59 ± 0.05										1.74 ± 0.44
180	48.39	1879	3-Methyl-cyclohexene	Hydrocarbons	C_7H_{12}							0.66 ± 0.06				
181	48.401	1879	2-Methoxy-3-(1-methylpropyl) pyrazine	Nitrogen compounds	$C_9H_{14}N_2O$	0.20 ± 0.05										
182	48.486	1881	Methyl allylthioacetate	Organosulfur compounds	$C_6H_{10}O_2S$						0.65 ± 0.16					
183	48.872	1888	4-Hepten-1-ol	Alcohols	$C_7H_{14}O$							0.41 ± 0.05				
184	48.969	1889	2-Ethyl-5-methylfuran	Furanic compounds	$C_7H_{10}O$		0.57 ± 0.30									
185	49.167	1893	(E)-3-Nonen-2-one	Ketones	$C_9H_{16}O$							0.26 ± 0.02				
186	49.421	1898	Formic acid	Carboxylic acids	CH_2O_2	1.80 ± 0.07	12.7 ± 2.08		4.90 ± 0.77	22.3 ± 0.78						
187	49.579	1900	Benzaldehyde	Aldehydes	C_7H_6O					6.02 ± 1.62		13.5 ± 2.17	1.38 ± 0.06	0.92 ± 0.20	1.25 ± 0.50	0.43 ± 0.14

188	50.178	1912	4-Methylpentyl isothiocyanate	Organosulfur compounds	$C_7H_{13}NS$								6.60 ± 1.77			
189	51.057	1929	Propanoic acid	Carboxylic acids	$C_3H_6O_2$	0.21 ± 0.07	0.59 ± 0.05			1.20 ± 0.42					0.39 ± 0.03	
190	50.249	1914	2,5-Dimethylacetophenone	Ketones	$C_{10}H_{12}O$				0.37 ± 0.01							
191	50.389	1916	(E)-2-Nonenal	Aldehydes	$C_9H_{16}O$				4.88 ± 0.73	2.95 ± 0.34		0.17 ± 0.02			1.06 ± 0.05	
192	50.429	1917	(Z)-4-Decenal	Aldehydes	$C_{10}H_{18}O$										3.28 ± 0.40	
193	51.658	1941	6,6-Dimethyl-2-methylenebicyclo[2.2.1]heptan-3-one	Ketones	$C_{10}H_{14}O$					3.37 ± 0.48						
194	51.684	1942	Cyclooctane	Hydrocarbons	C_8H_{16}		0.32 ± 0.07									
195	51.732	1942	1-Octanol	Alcohols	$C_8H_{18}O$					0.54 ± 0.10		0.61 ± 0.13		0.19 ± 0.04	0.28 ± 0.08	0.41 ± 0.14
196	52.331	1954	3,5-Octadien-2-one	Ketones	$C_8H_{12}O$					2.83 ± 0.11		5.12 ± 0.64				
197	52.622	1959	5-Methylfurfural	Furanic compounds	$C_6H_6O_2$	15.8 ± 1.64	7.50 ± 3.74						0.24 ± 0.11	0.91 ± 0.69	0.94 ± 0.10	0.26 ± 0.06
198	52.754	1962	1,7,7-Trimethyl-bicyclo[2.2.1]hept-2-yl ester acetic acid	Esters	$C_{12}H_{20}O_2$				2.43 ± 0.53							
199	52.901	1965	Hexyl isothiocyanate	Organosulfur compounds	$C_7H_{13}NS$								2.09 ± 0.65			
200	52.955	1966	Isothymol methyl ether	Terpenic compounds	$C_{11}H_{16}O$				0.78 ± 0.05							
201	53.164	1969	3,5,5-Trimethyl-2-cyclohexen-1-one	Ketones	$C_9H_{14}O$							2.59 ± 0.32				
202	53.25	1971	2-Cyclopentene-1,4-dione	Ketones	$C_5H_4O_2$	0.50 ± 0.15	5.12 ± 1.81			4.92 ± 0.37					0.65 ± 0.10	
203	53.337	1973	2,4-Hexadien-1-ol	Alcohols	$C_6H_{10}O$									0.42 ± 0.07		
204	53.534	1976	6,6-Dimethylhepta-2,4-diene	Hydrocarbons	C_9H_{16}							1.41 ± 0.51				
205	53.712	1980	1,5-Diethenyl-3-methyl-2-methylene-(1α, 3α, 5α)-cyclohexane	Hydrocarbons	$C_{12}H_{18}$				1236.8 ± 143.4							
206	54.58	1995	(Z)-2-Octen-1-ol	Alcohols	$C_8H_{16}O$										0.15 ± 0.03	
207	54.597	1996	4,5,6,7-Tetrahydroindazole-3-spirocyclohexane	Nitrogen compounds	$C_{12}H_{18}N_2$				0.93 ± 0.14							
208	54.617	1996	β-Cyclocitral	Terpenic compounds	$C_{10}H_{16}O$							1.14 ± 0.37	1.92 ± 0.58	0.67 ± 0.11	0.48 ± 0.09	0.85 ± 0.09
209	54.698	1998	2,2-Dimethyl-3-octanol	Alcohols	$C_{10}H_{22}O$									0.93 ± 0.20		
210	54.766	1999	Myrtenal	Terpenic compounds	$C_{10}H_{14}O$					4.38 ± 1.28						
211	54.776	1999	2-(2-Propenyl)furan	Furanic compounds	C_7H_8O							0.36 ± 0.16				
212	54.888	2101	2-(2-Ethoxyethoxy)ethanol	Ethers	$C_6H_{14}O_3$	0.33 ± 0.06	0.26 ± 0.01		0.38 ± 0.07		1.04 ± 0.21		0.19 ± 0.01		0.22 ± 0.03	
213	54.941	2102	Ethyl 2-furancarboxylate	Furanic compounds	$C_7H_8O_3$											0.34 ± 0.01
214	55.284	2110	Butyrolactone	Ketones	$C_4H_6O_2$	0.88 ± 0.31	0.67 ± 0.04		1.14 ± 0.36	6.70 ± 1.89		0.32 ± 0.11	0.30 ± 0.09	0.34 ± 0.17	0.99 ± 0.10	2.72 ± 1.00

215	55.552	2115	2-Dodecenal	Aldehydes	$C_{12}H_{22}O$										0.37 ± 0.09	
216	55.656	2118	(E)-2-Decenal	Aldehydes	$C_{10}H_{18}O$					0.93 ± 0.06						
217	55.727	2119	1,7,7-Trimethyl-2-vinylbicyclo[2.2.1]hept-2-ene	Terpenic compounds	$C_{12}H_{18}$	0.19 ± 0.05										
218	55.819	2121	2-Propenoic acid	Carboxylic acids	$C_3H_4O_2$	1.87 ± 0.53										
219	55.967	2124	Benzeneacetaldehyde	Aldehydes	C_8H_8O								2.14 ± 0.12	1.29 ± 0.59	0.93 ± 0.27	
220	56.213	2129	Acetophenone	Ketones	C_8H_8O							3.57 ± 0.02				
221	56.268	2131	(E)-Pinocarveol	Terpenic compounds	$C_{10}H_{16}O$					8.37 ± 1.73						
222	56.39	2133	1,6-Octadien-3-ol, 4,7-dimethyl-isovalerate	Terpenic compounds	$C_{15}H_{26}O_2$	1.86 ± 0.63										
223	56.484	2135	Dipropyl trisulfide	Organosulfur compounds	$C_6H_{14}S_3$			29.1 ± 2.36								
224	56.634	2138	1-Decene	Hydrocarbons	$C_{10}H_{20}$									0.22 ± 0.10		0.59 ± 0.14
225	56.707	2140	(Z)-β-Farnesene	Terpenic compounds	$C_{15}H_{24}$	0.30 ± 0.11										
226	56.798	2142	α-Ocimene	Terpenic compounds	$C_{10}H_{16}$				145.0 ± 10.67							
227	56.878	2143	5-Bromo-1-hexene	Halogenated compounds	$C_6H_{11}Br$									4.49 ± 0.59		
228	56.918	2144	2-Furanmethanol	Furanic compounds	$C_5H_6O_2$	5.88 ± 1.04	9.21 ± 0.93	0.83 ± 0.37		43.3 ± 7.21			0.08 ± 0.03		2.63 ± 0.40	1.07 ± 0.11
229	56.981	2146	Linalyl isobutyrate	Esters	$C_{14}H_{24}O_2$				50.0 ± 7.47							
230	57.29	2152	3-Methylbutanoic acid	Carboxylic acids	$C_5H_{10}O_2$										0.11 ± 0.03	
231	57.394	2154	β-Citral	Terpenic compounds	$C_{10}H_{16}O$										0.49 ± 0.11	
232	57.149	2149	(E)-β-Farnesene	Terpenic compounds	$C_{15}H_{24}$				22.3 ± 3.23							0.29 ± 0.04
233	57.627	2159	5-Methyl-2(5H)-furanone	Furanic compounds	$C_5H_6O_2$		0.50 ± 0.10								0.24 ± 0.03	
234	57.746	2161	(Z)-3-Nonen-1-ol	Alcohols	$C_9H_{18}O$							0.64 ± 0.01				
235	57.819	2163	(E)-2-Hexenyl tiglate	Esters	$C_{11}H_{18}O_2$									6.43 ± 0.20		
236	57.97	2166	1-Isothiocyanato-heptane	Organosulfur compounds	$C_8H_{15}NS$								2.30 ± 0.72			
237	58.074	2168	1,3-Diisopropenyl-6-methylcyclohexene	Terpenic compounds	$C_{13}H_{20}$				5.33 ± 0.90	5.69 ± 1.27						
238	58.168	2170	α-Terpineol	Terpenic compounds	$C_{10}H_{18}O$											29.3 ± 0.42
239	58.178	2170	Crotonic acid	Carboxylic acids	$C_4H_6O_2$		0.46 ± 0.03									
240	58.344	2173	(E,E)-2,4-Nonadienal	Aldehydes	$C_9H_{14}O$					4.37 ± 1.42		0.51 ± 0.17			0.24 ± 0.06	
241	59.362	2194	trans-α-Bergamotene	Terpenic compounds	$C_{15}H_{24}$	0.31 ± 0.06			10.3 ± 1.40							
242	59.503	2197	5-Methyl-2-furanmethanol	Furanic compounds	$C_6H_8O_2$					0.31 ± 0.14						

243	59.594	2199	Geranial	Terpenic compounds	$C_{10}H_{16}O$										1.95 ± 0.30	
244	59.655	2200	1,2-Dimethoxybenzene	Ethers	$C_8H_{10}O_2$							3.24 ± 0.16		0.07 ± 0.03		
245	59.839	2204	3-Vinyl-1,2-dithiacyclohex-4-ene	Organosulfur compounds	$C_6H_8S_2$						138.5 ± 9.13					
246	59.891	2205	Naphthalene	Naphthalene derivatives	$C_{10}H_8$	0.28 ± 0.03				1.28 ± 0.35			0.47 ± 0.10	0.98 ± 0.20	0.11 ± 0.03	0.25 ± 0.05
247	59.752	2202	3,5-Dimethyl-2-(methylthio)thiophene	Organosulfur compounds	$C_7H_{10}S_2$		0.64 ± 0.22									
248	60.226	2212	2-Formylmethyl-4,6,6-trimethyl-bicyclo[3.1.1]hept-3-ene	Aldehydes	$C_{12}H_{18}O$				4.91 ± 1.70							
249	60.463	2217	1-Isothiocyanato-octane	Organosulfur compounds	$C_9H_{17}NS$								1.57 ± 0.77			
250	60.897	2227	2(5H)-Furanone	Furanic compounds	$C_4H_4O_2$	0.90 ± 0.24	1.39 ± 0.44			8.13 ± 1.50					0.19 ± 0.03	
251	61.073	2231	2-Vinyl-1,3-dithiane	Organosulfur compounds	$C_6H_{10}S_2$						3.07 ± 0.96					
252	61.131	2232	3,5-Diethyl-1,2,4-trithiolane	Organosulfur compounds	$C_6H_{12}S_3$		0.73 ± 0.14	10.6 ± 1.52								
253	61.274	2235	Santolina triene	Terpenic compounds	$C_{10}H_{16}$	1.46 ± 0.09			120.7 ± 17.2							
254	61.459	2239	1-(4-Methylphenyl)ethanone	Ketones	$C_9H_{10}O$					7.73 ± 1.67						
255	61.481	2239	1,3-Cyclopentanedione	Ketones	$C_5H_6O_2$	2.02 ± 0.19										2.65 ± 0.43
256	61.487	2240	2-Hydroxy-2-cyclopenten-1-one	Ketones	$C_5H_6O_2$		2.58 ± 0.39									
257	61.712	2244	5-Ethyl-2-imino-thiazolidin-4-one	Organosulfur compounds	$C_5H_8N_2OS$		0.23 ± 0.12									
258	61.763	2245	Methyl salicylate	Esters	$C_8H_8O_3$							0.15 ± 0.06	0.68 ± 0.26			
259	62.003	2250	1,2,4,5-Tetramethylbenzene	Benzene compounds	$C_{10}H_{14}$				0.99 ± 0.07							
260	62.2	2255	Diallyl trisulfide	Organosulfur compounds	$C_6H_{10}S_3$						215.2 ± 20.06					
261	62.513	2261	5-Hydroxymethylfurfural	Furanic compounds	$C_6H_6O_3$	64.3 ± 5.91			24.5 ± 1.97	113.1 ± 9.74					20.6 ± 0.10	
262	62.604	2263	1-Ethyl-2,4-dimethylbenzene	Benzene compounds	$C_{10}H_{14}$					1.25 ± 0.72						
263	62.681	2265	2,3-Dimethoxytoluene	Benzene compounds	$C_9H_{12}O_2$									0.30 ± 0.15		
264	62.701	2265	(E,E)-2,4-Decadienal	Aldehydes	$C_{10}H_{16}O$		0.67 ± 0.05	0.47 ± 0.06							1.69 ± 0.38	0.26 ± 0.05
265	62.795	2267	α-Ethynyl-benzenemethanol	Alcohols	C_9H_8O							0.16 ± 0.04				
266	62.817	2268	(Z)-Geraniol	Terpenic compounds	$C_{10}H_{18}O$				0.58 ± 0.04							
267	62.984	2271	Geosmin	Terpenic compounds	$C_{12}H_{22}O$	0.80 ± 0.01										
268	63.103	2274	3-Methyl-1,2-cyclopentanedione	Ketones	$C_6H_8O_2$					0.17 ± 0.03						
269	63.121	2274	2,2'-Bipyridine-3,3'-diol	Nitrogen compounds	$C_{10}H_8N_2O_2$	0.13 ± 0.03										

270	63.194	2276	1,2,3,4-Tetrahydro-1,6,8-trimethyl-naphthalene	Naphthalene derivatives	$C_{13}H_{18}$				0.60 ± 0.24					0.18 ± 0.10		
271	63.203	2276	(Z)-Carveol	Terpenic compounds	$C_{10}H_{16}O$					0.60 ± 0.31						0.26 ± 0.04
272	63.28	2277	2,3-Dimethyl-thiophene	Organosulfur compounds	C_6H_8S		4.50 ± 0.51									
273	63.402	2280	3-Vinyl-1,2-dithiacyclohex-5-ene	Organosulfur compounds	$C_6H_8S_2$						12.0 ± 2.30					
274	63.411	2280	Hexanoic acid	Carboxylic acids	$C_6H_{12}O_2$					1.93 ± 0.06					1.35 ± 0.30	0.89 ± 0.10
275	63.418	2280	Ethyl decanoate	Esters	$C_{12}H_{24}O_2$							1.69 ± 0.11				
276	63.487	2282	Geranyl acetone	Terpenic compounds	$C_{13}H_{22}O$			0.89 ± 0.37	1.32 ± 0.65						10.1 ± 0.10	3.91 ± 1.00
277	63.495	2282	p-Cimen-8-ol	Terpenic compounds	$C_{10}H_{14}O$	0.52 ± 0.06			1.36 ± 0.24	141.2 ± 14.22						
278	63.709	2286	2-Methoxy-phenol	Alcohols	$C_7H_8O_2$										0.74 ± 0.12	0.21 ± 0.03
279	63.79	2288	2,4-Dithiapentane	Organosulfur compounds	$C_3H_8S_2$						7.62 ± 1.74					
280	63.876	2290	2-(1,1-Dimethylethyl)-1,4-dimethoxy-benzene	Benzene compounds	$C_{12}H_{18}O_2$				1.14 ± 0.87							
281	63.969	2292	Benzyl alcohol	Alcohols	C_7H_8O							0.12 ± 0.02			0.30 ± 0.09	
282	64.209	2296	4-Methyl-5H-furan-2-one	Furanic compounds	C_7H_8O										0.11 ± 0.02	
283	64.316	2299	Linalyl isovalerate	Terpenic compounds	$C_{15}H_{26}O_2$				5.59 ± 1.40							
284	64.378	2300	2,4-Dimethyl-quinoline	Nitrogen compounds	$C_{11}H_{11}N$				4.81 ± 1.22							
285	64.525	2303	2,4-Dimethyl-cumene	Terpenic compounds	$C_{11}H_{16}$				0.90 ± 0.04							
286	64.535	2303	Phenylethyl alcohol	Alcohols	$C_8H_{10}O$							0.68 ± 0.02	3.19 ± 0.56	1.31 ± 0.59	2.60 ± 0.20	
287	64.701	2306	2-Phenylethyl cyanide	Nitrogen compounds	C_9H_9N								15.2 ± 4.76			
288	64.856	2309	Phenylacetonitrile	Nitrogen compounds	C_8H_7N							0.48 ± 0.15				
289	64.935	2311	β-Ionone	Terpenic compounds	$C_{13}H_{20}O$				0.72 ± 0.19			1.44 ± 0.06	5.87 ± 1.14	0.76 ± 0.69	0.58 ± 0.04	0.48 ± 0.06
290	65.363	2319	2,7-Dimethylnaphthalene	Naphthalene derivatives	$C_{12}H_{12}$				0.28 ± 0.14							
291	65.563	2323	2-Methoxy-5-methylthiophene	Organosulfur compounds	C_6H_8OS		1.33 ± 0.69									
292	65.579	2323	Citronellyl acetate	Terpenic compounds	$C_{12}H_{22}O_2$									0.36 ± 0.30		
293	65.649	2325	3,5-Dihydroxytoluene	Benzene compounds	$C_7H_8O_2$				4.88 ± 0.84							
294	65.649	2325	2,5-Furandicarboxaldehyde	Furanic compounds	$C_6H_4O_3$	1.36 ± 0.42	5.76 ± 1.31			4.18 ± 0.60					0.86 ± 0.11	
295	65.691	2326	3-Methylthiopropyl isothiocyanate	Organosulfur compounds	$C_5H_9NS_2$								0.94 ± 0.01			
296	65.825	2328	Dihydro-4-hydroxy-2-(3H)-furanone	Furanic compounds	$C_4H_6O_3$	2.67 ± 0.36										

297	65.917	2330	Phenol	Alcohols	C_6H_6O	0.23 ± 0.004						0.36 ± 0.08				
298	66.761	2346	Methyl cyclopropylcarboxylate	Esters	$C_5H_8O_2$						1.86 ± 0.51					
299	67.094	2352	1,3-Dihydroxy-2-propanone	Ketones	$C_3H_6O_3$		3.36 ± 0.60			7.43 ± 0.07						
300	67.261	2356	N-Methyl-1,3-propanediamine	Nitrogen compounds	$C_4H_{12}N_2$	6.81 ± 2.05				14.6 ± 4.62						
301	67.402	2358	2-Methyl-7-exo-phenyl-bicyclo[4.2.0]oct-1-ene	Terpenic compounds	$C_{15}H_{18}$								0.51 ± 0.15			
302	67.481	2360	(E)-4-Methoxy-2-hexene	Ethers	$C_7H_{14}O$					5.09 ± 1.17						
303	67.677	2363	Angelicine	Furanic compounds	$C_{11}H_6O_3$	0.65 ± 0.22										
304	68.029	2370	Carvacrol	Terpenic compounds	$C_{10}H_{14}O$				0.56 ± 0.10							
305	68.229	2374	Eugenol	Terpenic compounds	$C_{10}H_{12}O_2$										0.46 ± 0.08	3.36 ± 0.28
306	68.368	2376	Methylisoeugenol	Terpenic compounds	$C_{11}H_{14}O_2$											1.85 ± 0.42
307	68.587	2380	5-Acetoxymethyl-2-furaldehyde	Furanic compounds	$C_8H_8O_4$		2.56 ± 0.34			2.80 ± 0.17						
308	69.481	2397	Heptadecane	Hydrocarbons	$C_{17}H_{36}$				2.02 ± 0.09							
309	69.535	2398	2-Phenylethyl isothiocyanate	Organosulfur compounds	C_9H_9NS								2718.3 ± 190.4			
310	69.773	2402	Myristicin	Benzene compounds	$C_{11}H_{12}O_3$	3.80 ± 0.66				426.0 ± 33.37						1.13 ± 0.14
311	69.837	2403	2,3-Dihydro-3,5-dihydroxy--6-methyl-4H-pyran-4-one	Ketones	$C_6H_8O_4$		29.9 ± 2.25		10.3 ± 1.07							
312	70.306	2412	10,10-Dimethyl-2,6-dimethylenebicyclo[7.2.0]undecan-5-β-ol	Terpenic compounds	$C_{15}H_{24}O$				1.75 ± 0.54							
313	70.5	2415	Undecanoic acid	Carboxylic acids	$C_{11}H_{22}O_2$	10.4 ± 2.30				20.3 ± 9.58						
314	70.988	2424	3-Isothiocyanatopropyl-benzene	Organosulfur compounds	$C_{10}H_{11}NS$								3.08 ± 0.70			
315	70.99	2424	N-Methyl-N-nitroso-2-propanamine	Nitrogen compounds	$C_4H_{10}N_2O$					8.91 ± 1.29						
316	71.174	2427	(Z)-Isoeugenol	Terpenic compounds	$C_{10}H_{12}O_2$											0.41 ± 0.08
317	72.229	2446	(Z)-8-Dodecen-1-ol	Alcohols	$C_{12}H_{24}O$	13.1 ± 0.57										
318	72.802	2456	3-Furancarboxylic acid	Furanic compounds	$C_5H_4O_3$				1.84 ± 0.06							
319	72.929	2458	Benzenecarboxylic acid	Carboxylic acids	$C_7H_6O_2$							1.62 ± 0.04				
320	72.944	2459	2-Furancarboxylic acid	Furanic compounds	$C_5H_4O_3$		1.79 ± 0.33			5.91 ± 1.12						
Total volatile metabolites						61	49	29	59	71	21	88	51	57	77	65
Total Peak Area (×10[8])						4.62	3.42	6.72	38.31	27.84	17.31	5.73	28.16	2.86	2.88	8.68
% RSD (*n* = 3)						15.0	14.0	7.6	12.1	10.0	7.8	13.8	7.4	5.0	4.0	3.6
